# T_H_17, T_H_22 and T_Reg_ Cells Are Enriched in the Healthy Human Cecum

**DOI:** 10.1371/journal.pone.0041373

**Published:** 2012-07-19

**Authors:** Martin J. Wolff, Jacqueline M. Leung, Michael Davenport, Michael A. Poles, Ilseung Cho, P'ng Loke

**Affiliations:** 1 Division of Gastroenterology, Department of Medicine, New York University School of Medicine, New York, New York, United States of America; 2 Division of Medical Parasitology, Department of Microbiology, New York University School of Medicine, New York, New York, United States of America; 3 Division of Gastroenterology, Department of Medicine, Veterans Affairs New York Harbor Healthcare System, New York, New York, United States of America; Trinity College Dublin, Ireland

## Abstract

There is increasing evidence that dysregulation of CD4^+^ T cell populations leads to intestinal inflammation, but the regional distribution of these populations throughout the intestinal tract in healthy individuals remains unclear. Here, we show that T_H_17, T_H_22 and T_Reg_ cells are enriched in the healthy human cecum compared to the terminal ileum and sigmoid colon, whereas T_H_1 and T_H_2 cells do not significantly vary by location. Transcriptional profiling analysis of paired pinch biopsies from different regions of the intestine identified significant differences in the metabolic state of the terminal ileum, cecum, and sigmoid colon. An increased proportion of T_H_17 cells was positively associated with expression of resistin (RETN) and negatively associated with expression of trefoil factor 1 (TFF1). These results suggest that CD4^+^ T helper cells that are important in maintaining mucosal barrier function may be enriched in the cecum as a result of metabolic differences of the surrounding microenvironment.

## Introduction

The human intestinal epithelium represents a critical interface between our entire internal milieu and the outside world [1]. Appropriate mucosal homeostasis depends on interaction between the commensal microbiota, the intestinal epithelium, and the mucosal immune system [2]. Of mucosal immune cells, T_H_17 cells are particularly important in regulating intestinal immunity and have a complex role in human disease [1], [2]. For example, T_H_17 cells have been shown to be increased in active Crohn's disease [3] and are reduced in HIV infection [4]. Induced FoxP3^+^ regulatory T cells (T_Regs_) are developmentally linked to T_H_17 cells, with both requiring TGFβ for differentiation [5]. Recent evidence suggests that these two cell populations may arise from the same naïve precursor cells and exhibit plasticity [6]. For example, FoxP3^+^IL-17^+^ CD4^+^ cells have now been observed under several different inflammatory conditions [7], [8]. One of the cytokines produced by T_H_17 cells that is important in regulating mucosal barrier function is IL-22, which has been shown to promote epithelial proliferation and increase mucus production [9]. Not all T_H_17 cells produce IL-22, however, and a subset of CD4^+^ helper T cells that produce IL-22 but not IL-17 have been identified in humans and are termed T_H_22 cells [10]. While these different CD4^+^ T cell populations have been suggested to play important roles in regulating intestinal immunity, their exact functions in inflammation during human disease remain unclear, perhaps because an appropriate balance of these cells is required for healthy homeostasis.

In order to better understand how dysregulation among populations of CD4^+^ T cells in the intestinal lamina propria may be important during inflammatory conditions of the gastrointestinal tract, it is necessary to fully characterize these populations in healthy individuals. Specifically, the regional variations of these lymphocyte populations within the small and large intestine may provide important clues to their function *in vivo*. In order to address this question, we analyzed mucosal pinch biopsies collected from the terminal ileum (TI), ileocecal valve (ICV), appendiceal orifice (AO), and sigmoid colon (SC) from a healthy population presenting for screening colonoscopy. We were particularly interested in evaluating the cecal appendix given its implication in a number of inflammatory and infectious disease states, including ulcerative colitis and *Clostridium difficile* infection [11], [12]. Here we report that the proximal colon (cecum) is enriched in T_H_17, T_H_22, and T_Reg_ (but not T_H_1 or T_H_2) cell populations compared to the terminal ileum and distal large intestine in healthy individuals. We hypothesize that these differences are related to variations in metabolism and immune activation among different regions of the colon and small intestine.

## Results

### T_H_17 and T_Reg_ cells are enriched in the healthy human cecum

Different regions of the intestinal tract perform diverse dietary functions and are colonized with distinct concentrations of commensal bacteria [13]. We therefore hypothesized that there are important differences in the distribution of lymphocyte populations in the healthy human gut. Based on mounting evidence of the immunologic function of the human cecum [12], [14], our objective was to characterize the distribution of CD4^+^ T helper cell populations in the cecum relative to the terminal ileum and sigmoid colon in healthy individuals. We recruited 26 unique patients at average-risk for colon cancer who were undergoing screening colonoscopy and consented to participate in this study (Table 1). The majority of the subjects were black men with an average age of 58.9+6.2 (Table 1) and therefore may not be representative for other gender, age, and ethnic groups. Hematologic and renal function parameters were within normal range for the majority of subjects. As shown in Table 2, the prevalence of adenomatous polyp tissue obtained at colonoscopy in this cohort (30.7%) is typical of the average prevalence in the age-adjusted general population [15].

**Table 1:** Characteristics of the study population.

Variable	Mean (± SD) or Frequency (%)
Age, *y*	58.9±6.2
Sex, n (%)	
Female	2 (7.7)
Male	24 (92.3)
Body mass index (kg/m^2^)	29.3±3.3
Ethnicity (n, %)	
Caucasian	4 (15.4)
Black	18 (69.2)
Hispanic	3 (11.5)
Other	1 (3.8)
Laboratory parameters	
Hemoglobin (g/dL)	13.7±1.4
Hematocrit (%)	40.6±3.8
Mean corpuscular volume (fl)	90.5±6.5
Blood urea nitrogen (mg/dL)	14.8±3.8
Creatinine (mg/dL)	1.01±0.1
Previous colonoscopies, n (%)	
Yes	5 (19.2)
No	21 (80.8)

**Table 2:** Endoscopic findings and pathologic diagnoses at screening colonoscopy.

Variable	Mean (± SD) or Frequency (%)
Endoscopic findings, n (%)	
Normal	11 (42.3)
<3 polyps smaller than 1cm	12 (46.2)
≥3 polyps smaller than 1cm	0 (0)
Polyp(s) ≥1cm in size	3 (11.5)
Mass or tumor	0 (0)
Pathologic findings, n (%)	
No clinically indicated biopsies taken	12 (46.2)
Normal mucosa or hyperplastic polyp	6 (23.1)
Tubular adenoma	8 (30.7)
Tubulovillous or villous adenoma	0 (0)
Any polyp with high-grade dysplasia	0 (0)
Carcinoma	0 (0)

**Figure 1:**
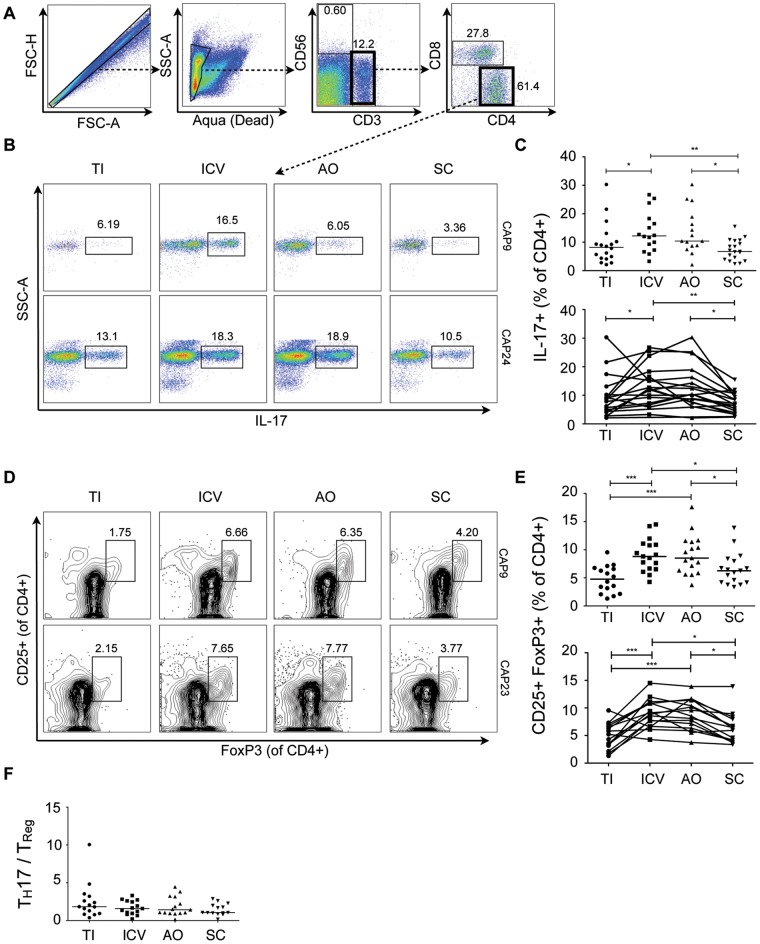
T_H_17 and T_Reg_ cells are enriched in the healthy human cecum. A) Representative gating strategy for the FACS analysis of lamina propria mononuclear cells from pinch biopsies obtained from four regions of healthy colonic mucosa. B) Representative FACS plots for two subjects (CAP9 and CAP24) showing IL-17^+^ cells (gated on CD4^+^ cells) from four biopsy locations in each subject. C) Cumulative data of IL-17^+^ CD4^+^ cells for all subjects. The top panel includes data from samples from all subjects with available flow cytometry data (N = 19) whereas the bottom panel shows data from subjects who have a complete set of paired samples from all four biopsy locations (N = 15). D) Representative FACS plots for two subjects (CAP9 and CAP23) showing CD25^+^FoxP3^+^ cells (gated on CD4^+^ cells). E) Cumulative data of CD25^+^FoxP3^+^ cells for all subjects. F) T_H_17/T_Reg_ ratios were plotted for all subjects according to biopsy location. Unless otherwise indicated, differences were not significant. *P<0.05; **P<0.01; ***P<0.001 (two-tailed Mann-Whitney). TI: Terminal Ileum, ICV: Ileocecal Valve, AO: Appendiceal Orifice, SC: Sigmoid Colon.

To assess regional differences in CD4^+^ T helper cell populations, lamina propria mononuclear cells (LPMCs) were isolated from pinch biopsies obtained at the terminal ileum (TI), ileocecal valve (ICV), appendiceal orifice (AO), and the sigmoid colon (SC). The ICV and AO are anatomical landmarks of the most proximal portion of the large intestine (i.e. the cecum). We were particularly interested in comparing immune parameters of the cecum with the TI because it is a critical site of luminal sampling and is rich in mucosal associated lymphoid tissue [16]. Biopsies were additionally taken from the SC (defined arbitrarily as 20cm from the anal verge in all subjects), because it represents a hindgut-derived distal site in the human colon that is easily accessible in all subjects. Biopsy-derived LPMCs were stimulated with phorbol 12- myristate 13-acetate (PMA) and ionomycin and then assessed for a number of immune markers by polychromatic flow cytometry. The flow cytometry panels used for this analysis are shown in Tables 3 and 4. By gating on singlet, live, CD3^+^ CD4^+^ T cells (Figure 1A), we compared the frequency of IL-17 producing CD4^+^ cells among different regions of the small and large intestine (Figure 1B). There was a significantly elevated frequency (Figure 1C) of IL-17 producing CD4^+^ cells in both parts of the cecum including the ICV (Median  = 12.2%) and AO (10.4%), compared to the TI (8.2%) and SC (6.7%). This was somewhat unexpected since T_H_17 cells have previously been reported to be enriched in the terminal ileum of mice [16], [17].

**Table 3:** Cytokine staining panel.

	Antigen	Clone	Manufacturer	Dilution
APC-Cy7	CD3	SP34-2	BD Pharmingen	100
PE-TR	CD4	MHCD0417	Invitrogen	75
A700	CD8	OKT8	eBioscience	75
PerCP	CD56	MEM-188	Biolegend	100
Pacific Blue	IFNγ	4S.B3	eBioscience	100
A488	IL-4	MP4-25D2	Invitrogen	50
APC	IL-17	eBio64CAP17	eBioscience	50
PE	IL-22	14292B	R&D Systems	50
PECy7	TNFα	MAb11	BD Pharmingen	100
AmCyan (AQUA)	Live/Dead		Invitrogen	60

**Table 4:** Nuclear antigen staining panel.

	Antigen	Clone	Manufacturer	Dilution
APC-Cy7	CD3	SP34-2	BD Pharmingen	100
PE-TR	CD4	MHCD0417	Invitrogen	75
A700	CD8	OKT8	eBioscience	75
PerCP	CD56	MEM-188	Biolegend	100
Pacific Blue	IL-17	BL168	Biolegend	50
APC	FoxP3	PCH101	eBioscience	50
PECy7	CD25	M-A251	BD Pharmingen	50
AmCyan (AQUA)	Live/Dead		Invitrogen	60

**Figure 2:**
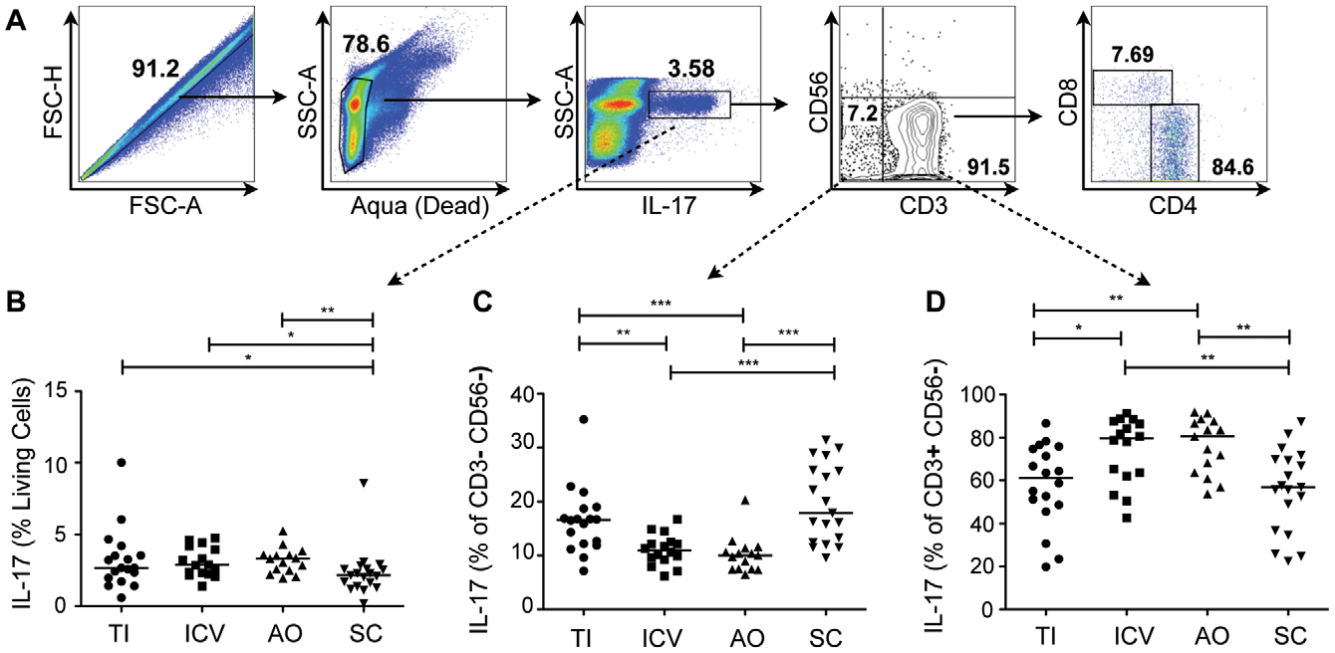
Non-CD4^+^ sources of IL-17 vary by biopsy location. (A) Representative gating strategy showing total singlet, live, and IL-17+ cells from the appendiceal orifice of one patient. IL-17^+^ cells were further gated into either CD3^−^ CD56^−^ cells or CD3^+^CD56^−^ cells. Approximately 80% of the CD3^+^ cells producing IL-17 are CD4^+^ lymphocytes and the remainder are mostly CD8^+^ lymphocytes (data not shown). (B) Plot of the total proportion of IL-17 producing cells, for all subjects, as a percentage of single, live (Aqua negative) cells. (C) Plot showing the proportion of CD3^−^ CD56^−^ cells that are producing IL-17. (D) Plot showing the corresponding proportion of CD3^+^ CD56^−^ cells that are producing IL-17. *P<0.05; **P<0.01; ***P<0.001.

To determine if there were significant non-CD4^+^ sources of IL-17 such as innate lymphocytes [18], we modified our gating strategy to first select for all of the IL-17^+^ cells (Figure 2A) in the live cell gate. Overall, there was an almost equivalent number of total IL-17^+^ cells among the different regions examined, with a slight decrease in the number of IL-17^+^ cells in the SC compared to the TI, ICV and AO (Figure 2B). While a large majority of all the IL-17^+^ cells were CD3^+^ (Figure 2A), there were also some CD3^-^CD56^-^ cells that were IL-17^+^ and these were significantly greater in numbers in the TI and SC (Figure 2C). This indicates a possible inverse relationship in the cecum between IL-17 production by CD3^−^ CD56^−^ cells and IL-17 production by CD3^+^ CD56^−^ cells, which were predominantly CD4^+^ (Figure 2A & 2B). Whereas there were more T_H_17 cells in the cecum, there may be more innate lymphocytes producing IL-17 in the TI and the SC.

Previous studies of mucosal biopsies from HIV infected individuals have indicated that increased frequency of T_H_17 cells may be accompanied by reduced frequencies of T_Regs_ because of the developmental link between these two populations [4]. However, when we assessed the frequency of CD4^+^CD25^+^FoxP3^+^ cells, we found that T_Regs_ were also significantly enriched in the cecum (Figure 1D & E). The proportion of CD25^+^FoxP3^+^ cells in the CD4^+^ compartment was significantly higher in the ICV (8.8%) and AO (8.5%) compared to the TI (4.8%) and SC (6.3%) (Figure 1D & E). These results indicate that T_H_17/T_Reg_ balance is maintained across different locations of the healthy human intestine. Though T_H_17 and T_Regs_ are enriched in the proximal colon, there were no significant differences in the regional variation of the ratio of T_H_17/T_Regs_ in healthy individuals (Figure 1F).

Because it was not possible to standardize the quantity of tissue that was processed for isolating LPMCs, the results here are presented as proportional data to total CD4^+^ cells. Absolute cell counts could vary based on variation in the size of processed pinch biopsies. CD4^+^ cell viability was relatively uniform across all locations, with the exception being a slight increase in CD4^+^ viability from the appendiceal orifice compared to the sigmoid colon (Table 5).

**Table 5:** CD4+ viability among biopsy locations.

	Terminal Ileum	Ileocecal Valve	Appendiceal Orifice	Sigmoid Colon
CD4^+^ Viability (%)	62.0±16.6	71.1±7.55	73.4±1.98	61.4±11.3

Mean and standard deviations are shown above.

Only comparison of CD4^+^ viability between the appendiceal orifice and sigmoid colon was found to be statistically significant.

### T_H_22 and IL-17 producing T_Reg_ cells are also enriched in the healthy human cecum

**Figure 3:**
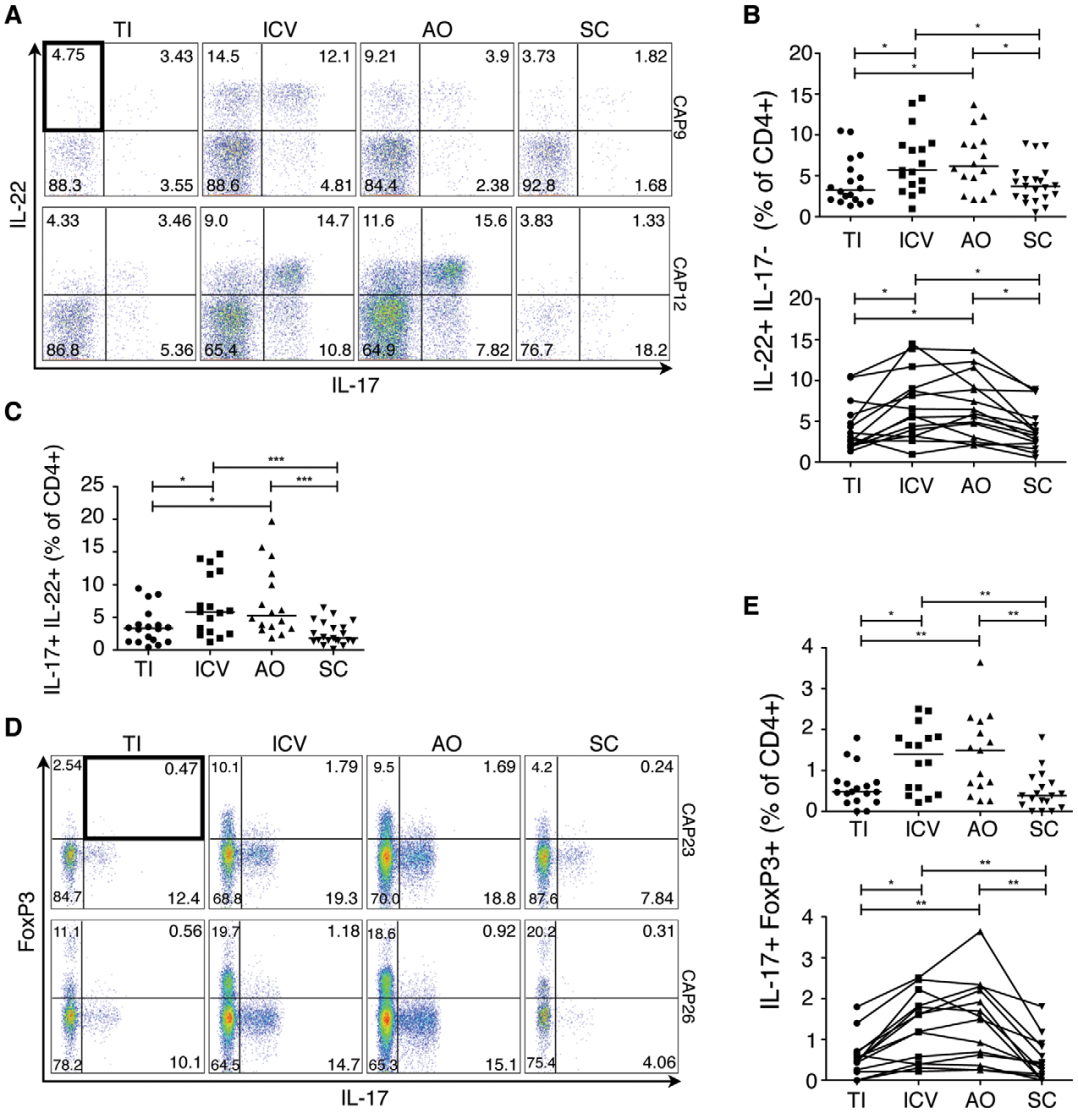
T_H_22 and IL-17^+^FoxP3^+^ cells are enriched in the healthy human cecum. A) Representative FACS plots for two subjects (CAP9 and CAP12) showing intracellular cytokine staining for IL-17 and IL-22 of lamina propria CD4^+^ cells. B) Cumulative data of IL-22^+^ IL-17^−^ CD4^+^ cells for all subjects. The top panel includes data from all samples from all subjects (N = 19) whereas the bottom panel shows data from subjects (N = 15) who have a complete set of paired samples from all four biopsy locations. C) Representative FACS plots for two subjects (CAP23 and CAP26) showing staining for IL-17^+^FoxP3^+^ lamina propria CD4^+^ cells. D) Cumulative data of IL-17^+^FoxP3^+^CD4^+^ cells for all subjects. Unless otherwise indicated, differences were not significant. *P<0.05; **P<0.01. TI: Terminal Ileum, ICV: Ileocecal Valve, AO: Appendiceal Orifice, SC: Sigmoid Colon.

Recently, a population of CD4^+^ helper T cells that produces IL-22 but not IL-17 has been identified [10] and termed T_H_22 cells. We previously found that IL-22 producing CD4^+^ cells were associated with helminth infection and disease remission in a patient with ulcerative colitis [19]. However, the normal variation and distribution of T_H_22 cells across the intestine under baseline conditions is not known. In this study, we found that CD4^+^IL-22^+^IL17^−^ T cells were also significantly elevated in the ICV (5.7%) and AO (5.9%), compared to the TI (3.1%) and the SC (3.6%), mirroring the results from T_H_17 and T_Reg_ cells (Figure 3A & 3B). Since IL-22 is important in controlling bacterial infections in the gut, such as *Citrobacter rodentium* [9], the enrichment of T_H_22 as well as T_H_17 cells in the cecum may indicate the presence of potentially pathogenic bacteria in this region that are actively restrained by the mucosal immune response. Additionally, IL-22 producing T_H_17 cells were also enriched in the cecum (Figure 3C).

Finally, we examined the distribution of IL-17 producing T_Regs_, which have been reported to be elevated in the inflamed mucosa of Crohn's disease patients [7] as well as ulcerative colitis patients [8]. We found that CD4^+^IL-17^+^FoxP3^+^ cells showed a similar pattern to T_H_17, T_H_22 and T_Regs_, being significantly elevated in the ICV (1.4%) and the AO (1.5%) compared to the TI (0.5%) and the SC (0.4%) (Figure 3D & 3E).

**Figure 4:**
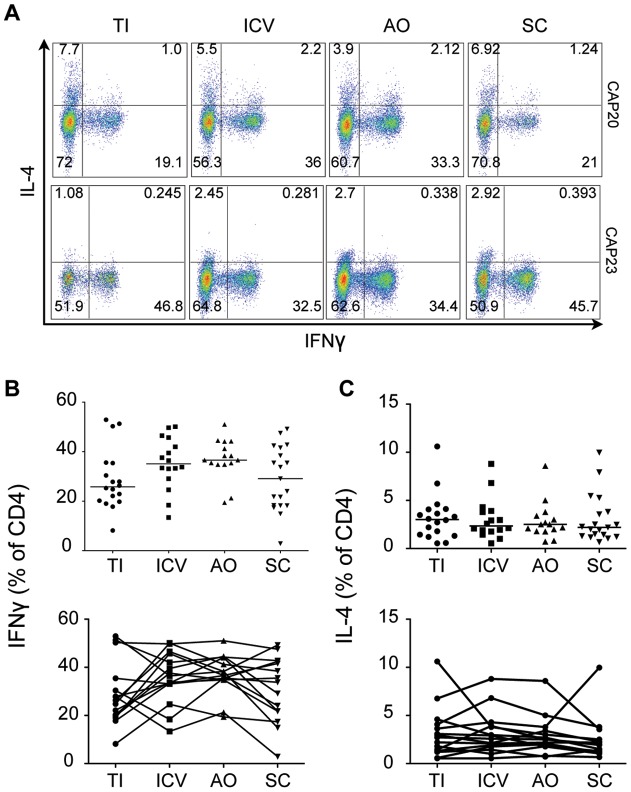
T_H_1 and T_H_2 lymphocyte populations do not vary by region. A) Representative gating strategy showing IFNγ and IL-4 staining of CD4^+^ cells isolated from four regions of the intestine in two subjects (CAP 20 and CAP23). B) IFNγ and C) IL-4 production by CD4^+^ isolated from each region for all subjects. Differences between regions are not statistically significant (two tailed Mann-Whitney). TI: Terminal Ileum, ICV: Ileocecal Valve, AO: Appendiceal Orifice, SC: Sigmoid Colon.

Together, these results from lamina propria CD4^+^ T cells suggest that there may be more immune activation in the cecum compared to both the small intestine (TI) and the distal colon (SC). However, only those CD4^+^ cell populations that have been reported to be important for mucosal homeostasis were enriched in the cecum. When we examined T_H_1 and T_H_2 populations that produce IFNγ and IL-4, there were no significant differences among the different sites that were biopsied in this study (Figure 4).

### Polycytokine production in lamina propria CD4^+^ T cells within the intestine

**Figure 5:**
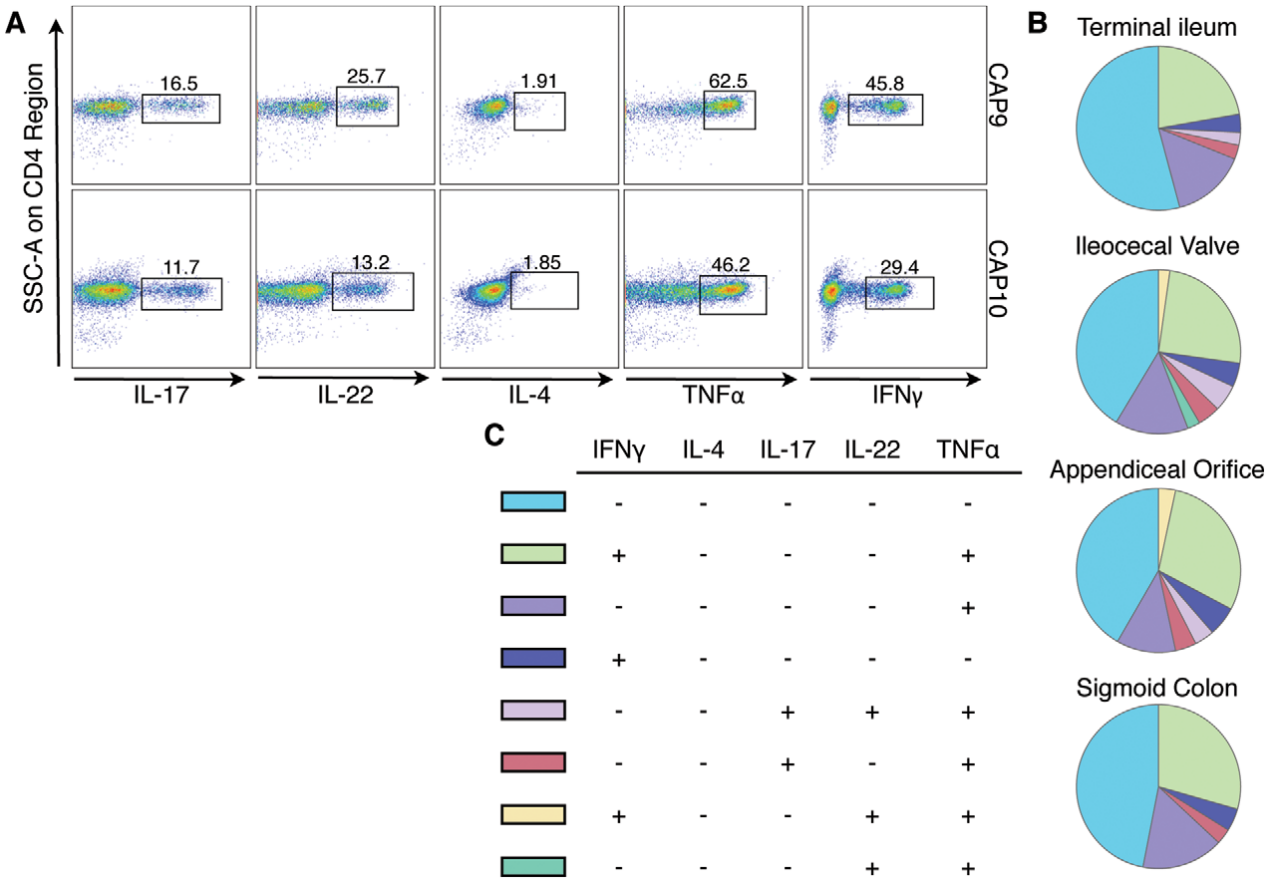
Polycytokine producing CD4^+^ cells analyzed by Boolean gate analysis from different regions of the intestinal tract. A) Boolean gates on IL-17, IL-22, IL-4, TNFα and IFNγ positive lamina propria CD4^+^ cells. Shown are representative FACS plots gated on CD4^+^ cells from the ileocecal valve of two subjects (CAP9 and CAP10). B) Pie charts showing the averaged pattern of cytokine production in CD4^+^ cells from the different regions of the gut (from N = 22 subjects). Each slice within the pie chart represents a specific combination of cytokine staining (denoted in C). Data is shown only for combinations with frequencies >2% of the total CD4^+^ population. IL-22^+^ TNFα^+^ IFNγ^+^ cells are >2% of CD4^+^ cells only from the ileocecal valve and appendiceal orifice. The permutation test at 10,000 permutations did not demonstrate significant differences among the pie charts represented.

We next analyzed the different combinations of cytokines produced by CD4^+^ T cell populations among different regions of the gut. We utilized Boolean gate analysis (Figure 5A) to expand our five-cytokine measurements (IL-17, IL-22, IL-4, TNFα and IFNγ) into 32 unique functional combinations of cytokine-producing CD4^+^ cells. The pie charts shown in Figure 5B illustrate the averaged overall pattern of cytokine production among lamina propria CD4^+^ cells in the different regions of the gut. Each slice within the pie chart represents a specific combination of cytokine production (denoted in Figure 5C). Not surprisingly, the most common CD4^+^ cells are the ones not producing any of the five measured cytokines, followed by cells producing a combination of IFNγ and TNFα and then IFNγ and TNFα individually (Figure 5B, C). No significant differences in the composition of cytokine-producing CD4^+^ cells among the various biopsy locations were found using permutation testing in SPICE. However, we noted that populations of CD4^+^ cells producing IFNγ, TNFα, and IL-22 in combination, as well as TNFα and IL-22 together, appear to be more prevalent in the cecum compared to the terminal ileum and sigmoid colon.

### Transcriptional profiling analysis of biopsies from different regions of the intestine

**Figure 6:**
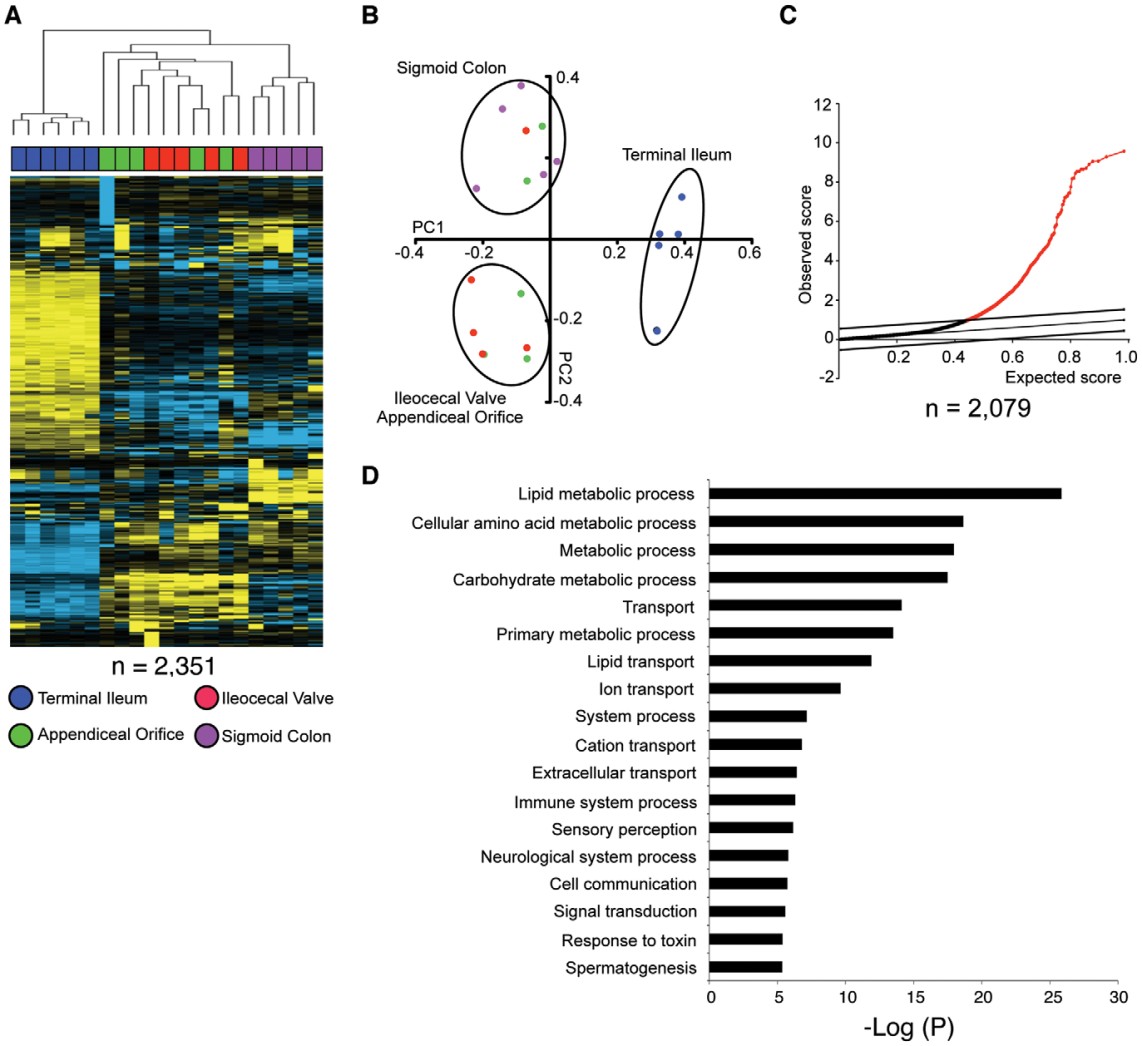
Transcriptional profiling analysis identifies regional variations of gene expression in the intestinal tract. A) Unsupervised hierarchical clustering analysis was used to organize gene probes and samples. Each row represents an individual gene probe and each column represents an individual sample. Black indicates the median level of expression; yellow, greater then median expression; blue, less than median expression. Horizontal bars at the top of the figure indicate the dispersal of samples according to biopsy location (blue: terminal ileum; red: ileocecal valve; green: appendiceal orifice; purple: sigmoid colon). Data was filtered for probes with expressions levels that vary by a standard deviation of at least 1.0 to yield n = 2,351 unique gene probes. B) Unsupervised principal component analysis showed segregation of terminal ileum samples from colon samples along PC1 and segregation of distal (sigmoid) from proximal colon (ileocecal valve and appendiceal orifice) along PC2. C) Multiclass statistical analysis of microarrays (SAM) identified 2,079 unique gene probes that vary significantly among the sites biopsied (FDR 0%). D) Gene ontology analysis of these gene probes was performed in order to classify genes according to biological processes. Of the 2,079 unique significant gene probes, 304 were classified as relating to the immune system.

To determine if the microenvironment from which we isolated lamina propria CD4^+^ cells may provide an explanation for the regional variation in the T_H_17, T_H_22 and T_Reg_ populations, we performed gene expression profiling experiments of paired pinch biopsies collected from the same regions that were analyzed by flow cytometry. RNA from a subset of matched samples (total N = 21, TI  = 6, ICV  = 5, AO  = 5, SC  = 5) from five subjects was isolated for DNA microarray analysis on the Agilent platform, in order to identify molecular signatures that may help to explain compositional differences in the CD4^+^ compartment. Unsupervised hierarchical clustering analysis was performed on 2,351 gene probes that were filtered to have a standard deviation of at least 1.0 between all 21 samples (Figure 6A). This analysis showed that the gene expression profiles of biopsies from the terminal ileum clustered very distinctly from biopsies of the cecum (including AO and ICV) and the sigmoid colon (SC). The appendiceal orifice and ileocecal valve were less distinct from each other and clustered together separately from the sigmoid colon and the terminal ileum. Principal component analysis of this dataset (Figure 6B) provided a similar picture, segregating the terminal ileum from the large intestine based on PC1, with the proximal colon (cecum, including AO and ICV) segregating from the sigmoid colon on PC2 (Figure 6B).

We then conducted a supervised analysis using multiclass statistical analysis of microarrays (SAM) to identify genes that were differentially expressed among regions of the intestine. 2,079 unique gene probes were identified at a False Discovery Rate (FDR) of 0% that varied significantly based on biopsy location (Figure 6C). Gene ontology (GO) analysis of this list of genes indicated that differences in lipid, protein, and carbohydrate metabolism varied most significantly among the different regions of the intestine that were biopsied (Figure 6D). Of note, 304 genes were grouped into the immunologic processes category (Table 6).

**Figure 7:**
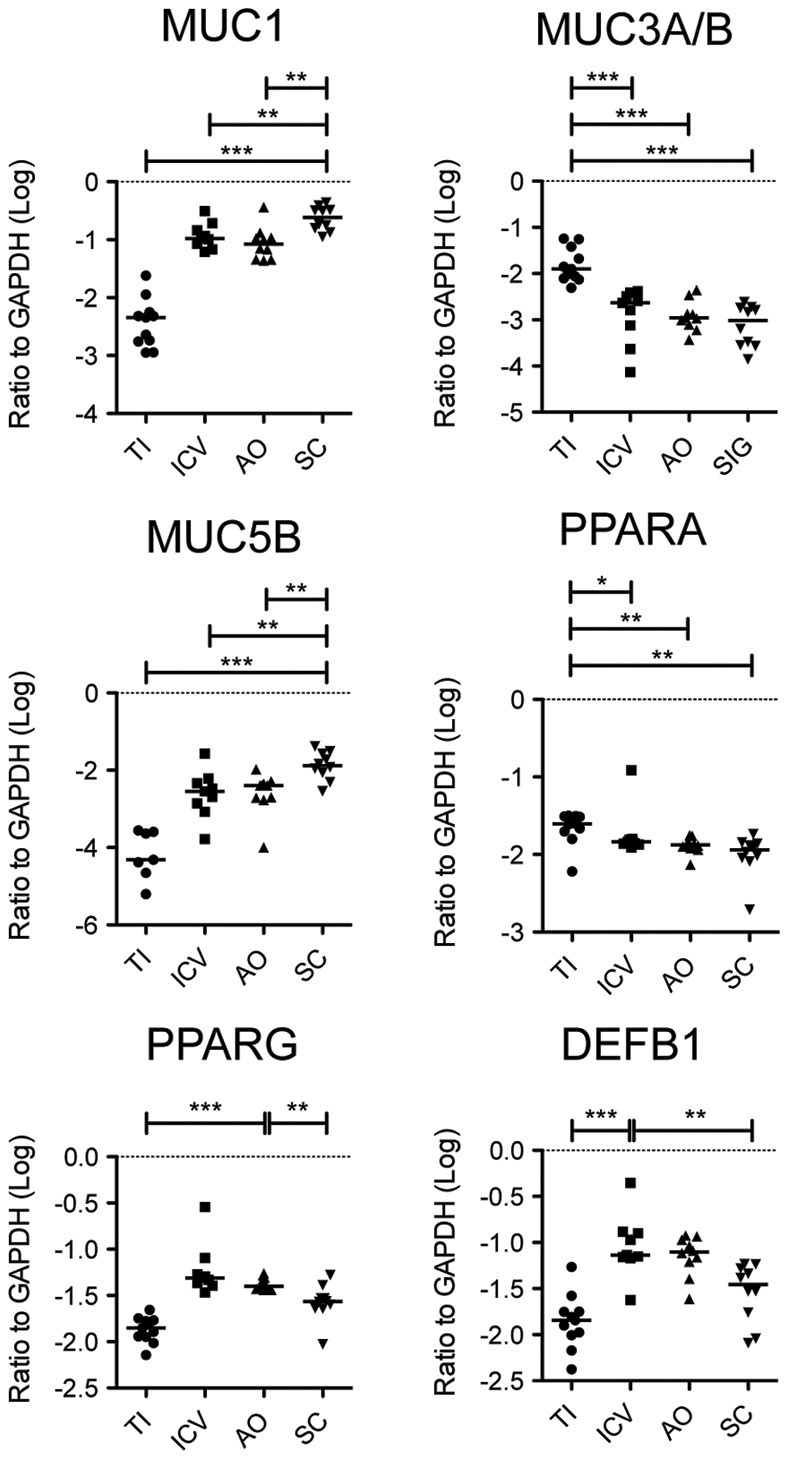
Verification of gene expression by real-time PCR analysis of intestinal biopsy samples. Expression levels of selected genes were measured in 40 intestinal biopsy samples from the terminal ileum (TI), ileocecal valve (ICV), appendiceal orifice (AO), and sigmoid colon (SC) and normalized to glyceraldehyde-3 dehydrogenase (GAPDH) transcript levels. Values were log transformed as shown. Horizontal bars indicate median expression levels. Statistical significance between groups was determined by two-tailed Mann Whitney test. *P<0.05; **P<0.01; ***P<0.001.

We were particularly interested in the differential expression of mucin genes among different regions of the gastrointestinal tract because of their important role in mucosal barrier function [20]. Furthermore, based on microarray analysis of an individual who treated his ulcerative colitis with *Trichuris trichiura,* we have hypothesized that increased mucus production could mitigate inflammatory pathology and bacterial attachment in the colon [19], [20]. We confirmed by real-time PCR on an expanded subset of samples (N = 40, TI  = 11, ICV  = 9, AO  = 10, SC  = 10) that MUC1 was most highly expressed in the sigmoid colon and least expressed in the terminal ileum, whereas MUC3 was most highly expressed in the terminal ileum and least expressed in the distal colon (Figure 7). MUC1 and MUC3 are membrane bound mucins. MUC1 is a tumor-associated molecule frequently overexpressed in carcinomas [21] and MUC3 is the major component of the glycocalyx of the small intestine [22]. Variants of MUC3 have previously been associated with inflammatory bowel diseases [23]. MUC5B had a similar expression profile to MUC1, being most elevated in the sigmoid colon (Figure 7), but is a secreted polymeric mucin that can be dysregulated in the lung of subjects with idiopathic pulmonary fibrosis [24].

**Table 6:** Differential expression of gene probes involved in immunologic processes.

Biopsy Location	Gene Probe (Immunologic Processes Only)
Terminal Ileum (n = 126)	CPO, PMP22, MS4A10, NTS, ABCG5, ABCG8, FBP1, ABCC2, HEBP1, DPP4, MAF, MEP1B, GSTA5, LIPA, GPX4, ENST00000377823, MSRA, SHBG, MUC3A, NLRP6, DAB1, PLB1, GSTA2, CD82, LMTK3, NPY6R, MOCOS, SEPP1, BPHL, MEP1A, CCRL1, ABCD1, CCL25, MOSC2, ABCC6, PRKAB2, ENST00000370725, SEMA6C, STAU2, CTSH, BAI2, ACSS1, TNFRSF10C, ANGPTL4, F10, CES2, TMPRSS7, CREB3L3, F11, PPARA, TNFSF15, DNAJB7, HTR4, ABCB1, ATOX1, LGALS2, HLA-DRA, NR1I3, NPNT, HLA-DMB, KLF7, ABHD6, ITGB8, JAG2, SEMA3B, TNFRSF10B, IGBP1, NCK2, GULP1, LRRC28, GPR128, PRSS7, CXCR7, CRIP1, SEMA3G, TESK2, NFATC3, RORC, CREB3L2, CD74, IFIT3, ABCG2, PLA2R1, TFPI, C8G, RAG1, LRRC66, MATN2, ERBB2IP, TMIGD1, KIR3DL2, CFB, NR1I2, TNC, ENST00000374707, ULK3, HLA-DMA, PPP1CC, CCR9, PTPRD, ADRB1, PON3, CD8B, JDP2, IRF8, PLS1, MAOA, DAPK2, CTSO, PRLR, EPHX2, PGCP, LMO4, LRRC40, TRIM32, MAP3K13, GBP3, SESTD1, CCK, PTPRH, PRKAG2, PTPRM, CD36, ABCC8, ETV7, ENST00000372411
Ileocecal Valve (n = 32)	ADORA2B, MOSC1, ETHE1, MB, HSF1, CTSL1, RSU1, COL8A1, CHP, PLAC8, TSPAN7, CD59, FGFR2, LGALS9C, EDA, PRDX1, NTRK2, KLK1, CLC, CCR10, F2RL1, SPR, TNFRSF17, CLIC1, PLAUR, TMPRSS4, LGALS4, FUT6, HSPG2, NLK, MFSD5, LRRC32
Appendiceal Orifice (n = 63)	CCL28, OASL, IER3, TSPAN1, CD9, PIGR, PPARG, FUT3, KLK3, KLHDC9, KLF8, SMAGP, LITAF, SPRYD4, IGHA2, IGHA1, LRG1, LIMK2, DRD5, TST, HSPB1, PRDX5, OSCP1, GPR39, ABCC3, C1QBP, NPY1R, ACSM3, PTGER2, FLRT3, GPR37L1, ABL1, NPY5R, LGALS9, RFXANK, ELF3, FAR2, KLK11, IGKC, ABCB11, SCAP, CPAMD8, DAO, NETO2, ABCC5, DDR1, SDC4, OAS1, CFI, AIFM3, COL9A3, MGST1, ABHD5, PLSCR1, ACSM1, ECSIT, DUSP21, RNF183, DEFB1, NCK1, PTPRA, CTSL2, GPR110
Sigmoid Colon (n = 83)	TFCP2L1, MUC1, LRFN4, EHF, VSIG2, CKB, SELENBP1, GPX2, CFTR, EPHA4, PNKD, EFEMP1, LRRN2, PRDX6, CAMK1D, GLIPR2, IL1R2, PPY, PPIF, GHR, SCARA5, FBLN2, IGFALS, NOX1, TK1, EPHB2, PTGDR, CFD, IGLC3, THBS1, CLIC4, CD14, TIMD4, IGF1R, ROR1, MUC5B, TRIO, GPR125, EPOR, PTPRU, C1QTNF6, ENST00000453184, GPR161, KLHDC4, NOTCH2, CFH, CFHR3, IGLL1, ENST00000390247, HSP90B1, EPHA3, KLK15, ENST00000390243, SEMA3F, NFASC, BATF2, ACR, CTSC, DCN, CDK4, IL1F7, PPIH, S100A10, DCBLD2, FKBP11, IGJ, CD81, FBLN1, PRDX4, LAMA2, RABEPK, CRYAB, FN1, STIL, NTRK3, LRIG3, SLIT3, TNFAIP6, ITPR2, FKBP9, SCRIB, RAD51, HSP90AB2P

**Figure 8:**
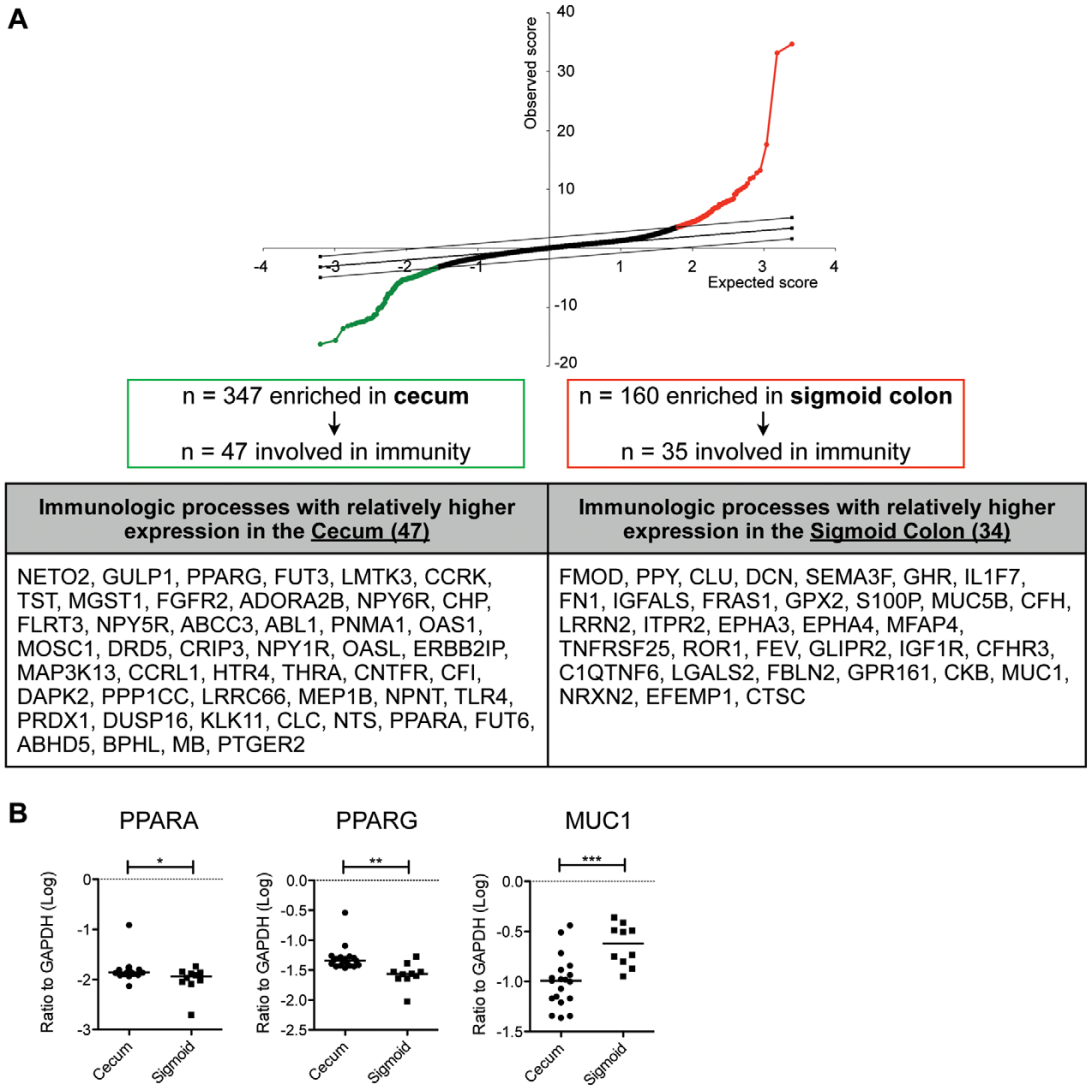
Identification of gene probes differentially expressed between the cecum (including ileocecal valve and appendiceal orifice) and the sigmoid colon by two-way SAM analysis. A) 347 gene probes were significantly elevated in the cecum relative to the sigmoid colon, 47 of which were involved in immunologic processes as determined by gene ontology analysis. 160 gene probes were significantly enriched in the sigmoid colon compared to the cecum, 35 of which were involved in immunologic processes. Genes probes related to immunologic processes are shown in the table below the figure. Analyses shown were performed at a FDR of 0%. B) Verification of gene expression by real-time PCR analysis of PPARA, PPARG, and MUC1 normalized to GAPDH transcript levels in 40 intestinal biopsy samples. Values were log transformed as shown. Horizontal bars indicate median expression levels. Statistical significance between groups was determined by two-tailed Mann Whitney tests. *P<0.05; **P<0.01; ***P<0.001.

We also explored the differential expression of the peroxisome proliferator-activated receptor (PPAR) members of the nuclear hormone receptor superfamily because they are important regulators of inflammation [25]. We confirmed that PPARα was more highly expressed in the terminal ileum (Figure 7), whereas PPARγ was more highly expressed in the cecum (AO and ICV). This was surprising because PPARγ has been reported to inhibit T_H_17 differentiation [26] whereas here we find that the cecum has relatively more T_H_17 cells as well as higher expression of PPARγ. Perhaps the anti-inflammatory effects of PPARγ also require enrichment in the cecum in order to balance higher levels of immune activation. We performed additional two-class analysis by comparing the proximal colon to the distal colon and excluding the terminal ileum, with similar results (Figure 8).

**Figure 9:**
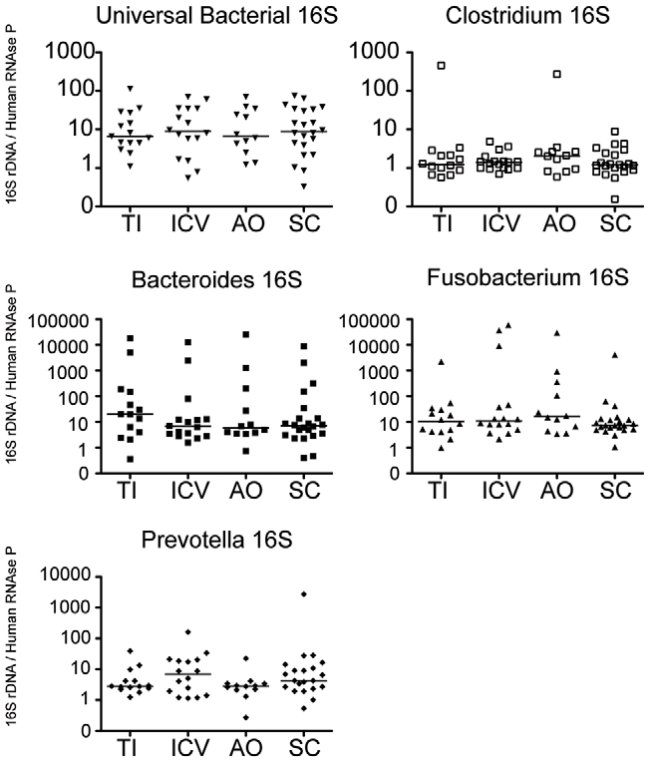
Bacterial attachment does not vary by biopsy location. Real-time PCR analysis of bacterial ribosomal 16S DNA sequences with primers specific for universal bacterial 16S, *Clostridiales*, *Bacteroides*, *Fusobacterium*, and *Prevotella* was compared to DNA copies of host human RNase P in order to calculate the relative bacterial density per biopsy sample relative to amount of host tissue. Differences between regions are not statistically significant (two tailed Mann-Whitney). TI: Terminal ileum, ICV: ileocecal valve, AO: appendiceal orifice, SC: sigmoid colon.

Consistent with the hypothesis that there is more bacterial mediated activation in the cecum, we confirmed elevated expression of the anti-microbial peptide DEFB1 (Figure 7). DEFB1 is the only defensin to be expressed in the uninflamed colon and certainly plays an important role in mucosal defense against microbes [27]. Although we speculated that the increased expression of DEFB1 and the enrichment of CD4^+^ T cells important in regulating mucosal barrier function may be driven by increased bacterial attachment to the intestinal wall in the cecum, we did not find significant differences in bacterial load present in these biopsies by qPCR analysis of bacterial specific ribosomal 16S DNA (Figure 9). Perhaps a more detailed deep sequencing analysis may identify specific differences in bacterial species that may be enriched on the intestinal wall of the cecum compared to other regions of the gastrointestinal tract.

### Correlating transcriptional profiling data with frequency of T_H_17 and T_Reg_ cells

**Figure 10:**
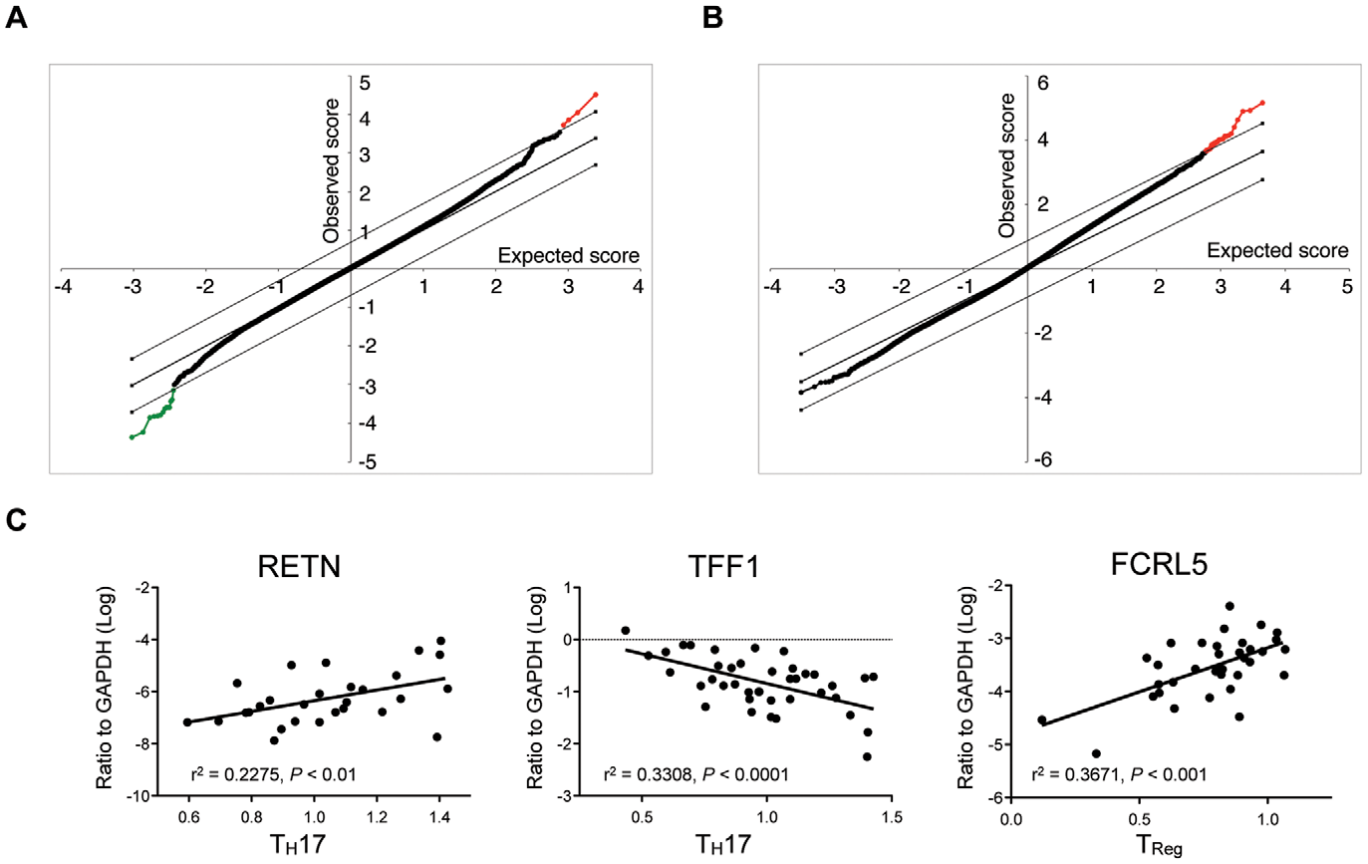
Identification of RETN, TFF1 and FCRL5 as genes with expression levels that correlate with frequencies of T_H_17 and T_Reg_ cells. (A) The frequency of T_H_17 cells was used as a quantitative variable to identify gene probes with correlated expression values using significance analysis of microarrays (SAM). Gene probes in red are positively correlated and gene probes in green are negatively correlated with T_H_17 cell frequency at a FDR of 0%. (B) Identification of gene probes correlated with frequency of CD4^+^CD25^+^FoxP3^+^ T_Reg_ cells. C) Real time PCR analysis of 40 biopsy samples confirms the positive correlation between T_H_17 cells with RETN expression, the negative correlation between T_H_17 cells with TFF1 expression, and the positive correlation between T_Reg_ cells with FCRL5 expression.

Having generated a combination of flow cytometry and transcriptional profiling datasets from paired biopsy samples, we sought to identify genes that have expression levels that are directly correlated with the frequency of lamina propria T_H_17 cells and T_Regs_ in these healthy individuals (Figure 10). We defined the frequency of T_H_17 cells and T_Regs_ obtained by flow cytometry as quantitative variable inputs to the statistical analysis of microarrays (SAM) algorithm in order to determine the strength of their relationship with gene expression values. Because of the relatively small number of samples, we used a conservative FDR of 0% as the cutoff for significance (Figure 10A–B and Table 7). For T_H_17 cells, we identified four gene probes that were positively correlated with T_H_17 cell frequency (including resistin (RETN)) and 12 gene probes that were negatively correlated with T_H_17 cell frequency (Figure 10A). Interestingly, many of the probes negatively associated with T_H_17 cell frequency encoded long non-coding RNA (lincRNA), for which the significance is unclear (Table 7). We did note with interest that trefoil factor 1 (TFF1) was among the most negatively associated genes with T_H_17 cell frequency. The positive association of RETN (r^2^ = 0.2275, *P* = <0.01) and negative association of TFF1 (r^2^ = 0.3308, *P* = <0.0001) with T_H_17 cell frequency were confirmed by RT-PCR analysis of additional biopsy samples (N = 40) from different regions of the intestine (Figure 10C). For T_Regs_ (CD4^+^CD25^+^FoxP3^+^ cells), we identified 22 gene probes that were positively correlated, but none that were negatively associated with T_Reg_ frequency (Figure 10B). Of these genes, Fc receptor-like protein 5 (FCRL5) was of particular interest because there was a strong association to T_Reg_ frequency and a closely related FCRL family member was reported to be associated with T_Reg_ function [28]. We confirmed by RT-PCR analysis that FCRL5 (r^2^ = 0.3671, *P* = <0.001) was indeed positively associated with T_Reg_ frequency (Figure 10C).

**Table 7:** Gene probes associated with Th17 and T-regulatory cell populations.

Cell Population	Positively Correlated Gene Probes	Negatively Correlated Gene Probes
T_H_17 (CD4^+^ IL-17^+^)	A_33_P3246244, FLJ38028, CELA3A, RETN	TFF1, lincRNA:chr3:195418384-195419180_F, lincRNA:chr3:195435000-195435680_F, lincRNA:chrX:73164173-73167168_F, HLA-DQB1, lincRNA:chrX:73164183-73214799_F, lincRNA:chrX:73164176-73167153_F, lincRNA:chrX:73164675-73232924_R, hg18:lincRNA:chr3:196920803-196924230_F, lincRNA:chr3:195436978-195437762_F, LOC729668, MXD1
T_Regs_ (CD4^+^ CD25^+^ FoxP3^+^)	CNR1, FCRL5, PRKD3, ZNF10, CD38, BCL2, lincRNA:chr2:157192429-157198954_R, ZDHHC2, UNC119B, ZNF783, TRAF5, ADAM28, LOC729678, CHD6, NCRNA00120, LDLRAD2, ARID5B, DAPL1, LOC100130298, NIN, KLF12, SUGT1L1	

## Discussion

In this study, we have systematically characterized regional differences of intestinal lamina propria CD4^+^ T cell populations by flow cytometry in a healthy population and identified gene expression patterns that correlate with their frequencies. We found the cecum to have significantly elevated frequencies of T_H_17, T_H_22, and T_Reg_ lymphocyte populations (but not T_H_1 or T_H_2 cells). Interestingly, these are the CD4^+^ subsets that are most important in regulating intestinal homeostasis. While T_H_17 cells are enriched in the terminal ileum in mice, we find here that the human cecum has a higher frequency of T_H_17 cells than does the ileum, reflecting important differences between mice and humans. Since intestinal mucosal CD4^+^ T cells are being increasingly studied under disease conditions (e.g. inflammatory bowel disease and HIV), it is important to rigorously investigate the baseline mucosal immune environment throughout the gut in healthy individuals. When mucosal biopsies are collected from different parts of the human intestine, there must be an awareness that inherent regional differences among CD4^+^ T cell populations are present in order to interpret study findings.

The results of our study are consistent with a previous histopathology study showing that the cecum is a site of inherent inflammation even in a normal healthy population without gastrointestinal symptoms [29]. Compared to the rectum, increased microscopic inflammation (especially greater cellularity of the lamina propria) in a significant proportion of cecal biopsies taken from endoscopically normal mucosa in healthy patients has been reported [29].

The cecum is generally considered to play a digestive role, being enlarged in herbivores to aid in the breakdown of cellulose [30]. However, there have been recent suggestions that it may have evolved in some species to perform an immunological function. In combination with the appendix, it has been proposed that the cecum could serve as a “safe house” for reconstituting commensal microbiota after an acute infectious diarrheal illness [30]. This hypothesis was partly driven by the finding that microbial biofilms are more abundant in the appendix than other parts of the human colon [13]. Secreted IgA and mucins in the cecum and appendix may support the growth of these microbial biofilms [13], [31]. More recently, it was reported that prior appendectomy was positively associated with increased risk of *Clostridium difficile* recurrence, suggesting that the appendix may play a protective role against infection. In contrast, previous appendectomy has been shown to inversely correlate with risk of colectomy, relapse rate, and extent of disease in ulcerative colitis (UC) patients in a series of case-control studies [32]. Prophylactic appendectomy has even been prospectively evaluated as a potential therapy for UC with promising results [11], [33]. Taken together, this literature suggests that microbial biofilms and immune activation in the cecum and appendix play a role in regulating homeostasis of intestinal microbiota, which could in turn impact homeostasis of the intestinal tissues. Based on this hypothesis, it would not be surprising that T_H_17, T_H_22, and T_Reg_ populations are enriched in the cecum to maintain an appropriate homeostasis between microbes and the intestinal mucosa.

Recently, innate lymphoid cells (ILCs) have emerged as an important non-CD4^+^ source of effector cytokines that may be particularly important at mucosal barrier surfaces [18]. IL-17 and IL-22 producing human ILCs are responsive to IL-23 signaling and could be important mediators of inflammatory bowel diseases [34]. Of additional interest was our observation that the increase in T_H_17 cells in the cecum (compared to the terminal ileum and sigmoid colon) was offset by having relatively fewer numbers of IL-17 producing CD3^−^CD56^−^ cells in this region. This inverse relationship raises the possibility of regulatory feedback between innate and adaptive sources of IL-17 in the healthy human intestinal tract.

We attempted to determine if the biopsies collected from the cecum had a greater bacterial load attached to the intestinal wall compared with those obtained from the terminal ileum and the sigmoid colon, but no significant differences were observed based on qPCR analysis of several bacterial ribosomal 16S DNA sequences (relative to host DNA). It is certainly conceivable that a deep sequencing analysis of bacterial ribosomal 16S units may lead to the identification of novel bacterial species or operational taxonomic units (OTUs) that are more abundant on the cecal wall compared to other parts of the intestine. Micro-heterogeneity between sample sites has been reported based on the very limited number of individuals who have been evaluated by deep sequencing studies of the colonic mucosa [35]–[37], but large variations in bacterial populations among intestinal regions has not been found. However, the cecum was not analyzed in detail in these studies. It would be extremely interesting to conduct deep sequencing analyses to determine the nature of the cecal microbiota attached to the mucosa using paired biopsy samples for which the gene expression profiles, as well as flow cytometry profiles of CD4^+^ T cell populations, is well characterized.

In a previous human study, gene expression profiling analysis was performed along the entire proximal-distal axis of the colon, but the terminal ileum was not included [38]. This study revealed two distinct patterns of transcript abundance along the proximal-distal axis of the large intestine. The colon was described as having a (i) dichotomous expression pattern consistent with its embryologic origins from both the midgut and the hindgut and hence segregating genes that are proximal or distal to a point that is two thirds the length of the transverse colon measured from the hepatic flexure and (ii) a gradual change in transcript levels from the cecum to the rectum, likely reflecting luminal flow and alterations in commensal microbiota. Our transcriptional profiling analysis of mucosal pinch biopsies confirms these results, with similar segregation of gene transcripts from both the proximal and distal colon and from the colon and terminal ileum. Additionally, we related transcriptional differences with the frequencies of CD4^+^ subsets along the intestine.

By correlating our flow cytometry analysis with gene expression profiling data from paired biopsy samples we discovered a positive relationship between the expression of resistin (RETN) and the frequency of T_H_17 cells. Resistin is a cytokine produced by adipose tissue and was originally discovered as a potential mediator of metabolic functions involved in insulin resistance [39]. More recently, it has been characterized as an inflammatory cytokine that is implicated in several pathologic intestinal conditions (e.g. colon cancer and inflammatory bowel disease) likely through induction of IL-1b, IL-6, IL-8, IL-12, TNFα, and other pro-inflammatory cytokines [40]. As a pro-inflammatory cytokine the positive association between the frequency of T_H_17 cells in the intestinal mucosa and expression levels of RETN is not surprising. PPARγ has been shown to regulate RETN activity in several studies [41], [42]. In this study we found that PPARγ expression and T_H_17 cell frequencies are both elevated in the human cecum, hence a relationship with RETN function is plausible. In addition, bacterial products like lipopolysaccharide (LPS) have been found to induce RETN expression in human and murine macrophages [43]. Future studies to establish a more mechanistic relationship between RETN and T_H_17 cells will need to be conducted before any conclusions can be made about the biological significance of this relationship.

We also found a negative relationship between the frequency of T_H_17 cells and the expression levels of TFF1. The trefoil factors are mucin-associated peptides associated with a number of human tissues, with TFF1 and TFF2 primarily located in the gastric mucosa and TFF3 predominantly present in the mucus cells of the small and large intestine [44]. Up-regulation of all three TFF members occurs at sites of mucosal injury and has been observed in patients with peptic ulcer disease and inflammatory bowel disease [44], [45]. TFF1 plays a critical role in mucosal protection and repair, with loss of TFF1 being associated with NF-κB-mediated inflammation and gastric carcinogenesis [46]. Based on these observations, it was surprising to observe a negative relationship between the frequencies of T_H_17 cells and TFF1, since both factors could be important for maintaining intestinal homeostasis. One possibility could be that the anti-microbial effects of TFF1 are acting through an independent pathway from T_H_17 cells. Hence, when bacterial loads are controlled through a TFF1 mediated pathway, fewer T_H_17 cells are required. This hypothesis could be tested further through the careful localization of TFF1 expression and presence of T_H_17 cells by immunohistochemistry of the intestinal tissues.

Finally, we found that T_Reg_ frequencies were positively associated with expression of FCRL5 (CD307). FCRL3, a closely related FCRL family member, is highly expressed on dysfunctional T_Regs_ that express high levels of PD-1 and have a memory phenotype [28]. While FCRL5 has mostly been characterized in B cells and B cell lymphomas [47], [48], polymorphisms have also been reported to be associated with autoimmune diseases such as ankylosing spondylitis [49], suggesting an immunoregulatory role [47]. The relationship between FCRL5 and T_Reg_ cells in regulating intestinal homeostasis warrants further investigation.

This study has important limitations. The majority of the enrolled subjects were older black men and so their mucosal environment may not be representative of younger patients in a disease state. Furthermore, complete colonoscopies requiring a preparative purge were performed to obtain biopsies from the cecum, a feature that may impact the analysis of the intestinal microenvironment. While the pinch biopsy samples obtained were approximately equal in size from all locations and from all subjects, we did not directly measure the absolute numbers of cells per gram of pinch biopsy tissue from each location. Importantly, CD4^+^ viability was relatively similar independent of biopsy location. Lastly, we recognize that differences in gene expression profiles of the pinch biopsies may be caused by heterogeneity of cellular populations and cellular density between samples. Nonetheless, this study is a step towards understanding location-specific variations in lamina propria CD4^+^ T cell populations in healthy individuals. This information may provide a basis for comparison when characterizing disease conditions that have characteristic distribution patterns along the intestinal tract, such as Crohn's disease and ulcerative colitis.

## Methods

### Ethics statement

New York Harbor Veterans Affairs Hospital Institutional Review Board approval was obtained before enrolling subjects in the study. Written informed consent was received from all participants.

### Research subjects

Consecutive subjects over the age of 50 who were at an average risk for colon cancer presented for screening colonoscopy and were enrolled in the study. Subjects were excluded who required endoscopy for an indication other than screening (e.g. family history of colon cancer, iron deficiency anemia, or hematochezia), were positive for human immunodeficiency virus (HIV), or who had absolute or relative contraindications to acquisition of biopsies for research purposes at the time of colonoscopy (e.g. systemic anticoagulation at the time of the procedure).

### Biopsy acquisition protocol

Six to eight pinch biopsies were obtained at colonoscopy from the terminal ileum (TI), the ileocecal valve (ICV), the appendiceal orifice (AO), and the sigmoid colon defined as 20 centimeters from the anal verge (SC) using sterile cold biopsy forceps. Not every location was biopsied in all subjects due to technical factors (e.g. a tortuous colon did not permit terminal ileum intubation). The mucosa was directly observed and irrigated after biopsy acquisition to ensure resolution of bleeding. One biopsy from each location was snap-frozen for RNA extraction in Trizol reagent (Invitrogen). The remaining biopsies were placed in complete RPMI containing 10% fetal calf serum, 0.05 mM 2-Mercaptoethanol, and penicillin/streptomycin/glutamine (Invitrogen).

### Isolation of lamina propria mononuclear cells

Biopsies were digested at 37°C for 1 hour in 100units/mL of collagenase Type VIII (Sigma) and 150 μg/mL DNase (Sigma) in complete RPMI. Cells were filtered through a 50 micron filter, washed with 5 mL 1X PBS, and pelleted. Cells were then resuspended in 40% Percoll (GE Healthcare), under-layered with 80% Percoll, and centrifuged at 2,200 rpm for 20 minutes at room temperature. Mononuclear cells were collected at the interface and used for subsequent flow cytometric analyses.

### FACS staining

Cells were stimulated with phorbol 12- myristate 13-acetate (PMA) and ionomycin for 4 hours at 37°C in the presence of brefeldin A (GolgiPlug, BD). Cells were subsequently divided for staining in one of two panels: (1) a “cytokine” panel whereby cells were stained with anti-CD3, anti-CD4, anti-CD8, and anti-CD56 and fixed in 4% paraformaldehyde in PBS and (2) a “nuclear antigen” panel whereby cells were stained with anti-CD3, anti-CD4, anti-CD8, anti-CD56, and anti-CD25 surface markers using the Fix/Perm Buffer Kit (eBioscience) for fixation and permeabilization. Live/Dead (aqua) viability dye (Invitrogen) was added to each panel. Cells in the “cytokine” panel were permeabilized and stained with an intracellular cytokine panel for interleukin (IL)-4, IL-17, IL-22, interferon-γ (IFNγ), and tumor necrosis factor-α (TNFα); cells in the “nuclear antigen” panel were stained with forkhead box P3 (FoxP3). Simultaneous multi-color flow cytometry was performed (BD LSR II). Results were analyzed in Flowjo (Treestar, Inc.) and statistical analysis was performed using Prism (GraphPad Software, Inc.)

### Boolean gate analysis of flow cytometric data

Five cytokine-producing CD4^+^ cell populations were analyzed in Flowjo using Boolean gates in order to calculate 32 possible cytokine populations. Data was exported to Simplified Presentation of Incredibly Complex Evaluations (SPICE) and filtered at a 2% cutoff to simplify the representation of the data (http://exon.niaid.nih.gov/spice/).

### Determination of bacterial attachment

DNA was isolated from intestinal pinch biopsies stored in Trizol using the DNeasy Blood and Tissue Kit (Qiagen) according to the manufacturer's instructions. After a chloroform purification and ethanol extraction step, DNA was probed with Taqman probes designed for bacterial genes of interest (Applied Biosystems). Bacterial probes were selected for universal bacteria 16S, Clostridiales, Bacteroides, Prevotella, and Fusobacterium as previously described [50]–[53]. The RNase P copy number assay was utilized as a basis to compare bacterial DNA content to host (human) DNA content (Applied Biosystems) by performing a logarithmic ratio of the cycle thresholds obtained for bacterial and host DNA.

### Transcriptional profiling and real-time PCR analysis of intestinal biopsies

Complementary DNA (cDNA) was reverse transcribed from RNA isolated from intestinal pinch biopsies according to manufacturer's instructions (Agilent Technologies). Cyanine 3-labeled, linearly amplified complementary RNA (cRNA) was then synthesized, purified, hybridized to microarray slides, and scanned using the High Resolution C Scanner according to the manufacturer's instructions (Agilent Technologies). The raw data was then uploaded to Microsoft Excel, where it was log-transformed quantile-normalized and median centered. Expression profiling data were filtered at a standard deviation of 1.0 before undergoing unsupervised hierarchical clustering analysis in Cluster (Eisenlab). Results were then visualized with Java Treeview. Principal component analysis (PCA) was performed on the gene expression parameters using Cluster to generate eigenvalues, and the resulting datasets were further compiled and plotted on Excel. Filtered data sets were also analyzed for statistically significant genes (either through unpaired two-way comparison or multiclass analysis) with the Statistical Analysis of Microarrays (SAM) software version 2.23 A (http://www-stat.stanford.edu/tibs/SAM). Correlations between gene expression data and flow cytometric parameters were performed using quantitative analysis in SAM. Gene ontology (GO) and pathway analyses were performed with Protein Analysis Through Evolutionary Relationships (PANTHER) software (http://www.pantherdb.org).

### Real-time quantitative PCR of intestinal biopsies

1μg of RNA from each sample was reverse-transcribed using SuperScript III Reverse Transcriptase (Invitrogen) and the resulting cDNA was used for quantitative real-time PCR with Taqman probes ordered directly from the manufacturer for the following genes of interest: DEFB1 (Hs00608345_m1), FCRL5 (Hs01070204_m1), MUC1 (Hs00159357_m1), MUC3 (Hs03649367_mH), MUC5 (Hs00861588_m1), PPARA (Hs00947539_m1), PPARG (Hs01115513_m1), TFF1 (Hs00907239_m1), and RETN (Hs00220767_m1) (Applied Biosystems). All cycle threshold values were normalized to GAPDH values. No significant differences in GAPDH expression were observed among the biopsy sites (data not shown). Data was analyzed with the t test using Prism 5.0 (GraphPad Software).

### Statistical analysis

Statistical analysis was performed using Prism 5.0 (GraphPad Software). The two-tailed Mann Whitney non-parametric t test was used to assess statistical significance for all samples.
